# Is There a Link between the Lipopolysaccharide of *Helicobacter pylori* Gastric MALT Lymphoma Associated Strains and Lymphoma Pathogenesis?

**DOI:** 10.1371/journal.pone.0007297

**Published:** 2009-10-06

**Authors:** Philippe Lehours, Zongli Zheng, Anna Skoglund, Francis Mégraud, Lars Engstrand

**Affiliations:** 1 INSERM, U853, Bordeaux, France; 2 Université Victor Segalen Bordeaux 2, Bordeaux, France; 3 CHU Bordeaux, National Reference Center for Helicobacters and Campylobacters, Pellegrin Hospital, Bordeaux, France; 4 Department of Medical Epidemiology and Biostatistics, Karolinska Institute Stockholm, Sweden; 5 Department of Microbiology, Tumor and Cell Biology, Karolinska Institute, Stockholm, Sweden; 6 Swedish Institute for Infectious Disease Control, Solna, Sweden; Charité-Universitätsmedizin Berlin, Germany

## Abstract

The aim of this study was to investigate the Lewis antigen expression in *Helicobacter pylori* gastric MALT lymphoma associated strains in comparison to chronic gastritis only strains. Forty MALT strains (19 *cag*PAI (−) and 21 *cag*PAI (+)) and 39 *cag*PAI frequency-matched gastritis strains (17 *cag*PAI (−) and 22 *cag*PAI (+)) were included in this study. The lipopolyssacharide for each strain was extracted using a hot phenol method and the expression of Le^x^ and Le^y^ were investigated using Western Blot. The data were analyzed according to the strains' *cag*PAI status and *vac*A genotype. Le^x^ was identified in 21 (52.5%) MALT strains and 29 (74.3%) gastritis strains. Le^y^ was identified in 30 (75%) MALT strains and 31 (79.5%) gastritis strains. There was an association between *cag*PAI positivity and Le^x^ expression among MALT strains (*p*<0.0001), but not in gastritis strains (*p* = 0.64). Among *cag*PAI (−) strains, isolates expressing solely Le^y^ were associated with MALT with an odds ratio of 64.2 (95% CI 4.9–841.0) when compared to strains expressing both Le^x^ and Le^y^. *vac*A genotypes did not modify the association between Lewis antigen expression and disease status. In conclusion, *cag*PAI (−) MALT strains have a particular Lewis antigen profile which could represent an adaptive mechanism to the host response or participate in MALT lymphomagenesis.

## Introduction

*Helicobacter pylori* was the first bacterium classified as a type I carcinogen (maximum level) [1]. Since its discovery, many research projects have focused on virulence factors or genetic markers but few studies have included *H. pylori* strains associated with gastric mucosa associated lymphoid tissue (MALT) lymphoma [2]–[5]. Gastric lymphoma is considered to be the classic lymphoma of MALT-type of the digestive tract [6]. It is a B lymphoma with a very unusual pathogenesis and evolution which evolves very progressively and stays localized in the stomach for a long time. The development of the lymphoma is directly linked to the *H. pylori* infection although it is not known why this evolution occurs in only a very small number of infected subjects. A large number of molecular events participating in the lymphomagenesis of MALT lymphomas have already been described, in which a chronic antigenic stimulation plays a pivotal role. Moreover, as the incidence of MALT lymphoma may correlate with different inflammatory cytokines and gene polymorphisms, the role of the host immune response has not been clearly defined yet [7].

The role of *H. pylori* strains on B-cell proliferation in low-grade MALT lymphoma is well established [8]–[10]. However, some questions remained unanswered. What is the nature of the *H. pylori* antigens recognized by lymphocytes? How does this recognition occur? Dendritic cells (DC) could play a role in antigen recognition and in inflammatory response in this disease. Indeed, the response of DC to a specific organism depends on the pathways activated in response to the microbial agent and to the cells present in the environment [11]. *H. pylori* lipopolyssacharide (LPS) is one of the key effectors of Toll Like Receptor-4 and it has been shown that the nature of the Lewis-type antigens expressed on the surface of the LPS of *H. pylori* determines the interaction with DC via the recognition by a C-type lectin called DC-SIGN at the surface of DC [12]. It has been also suggested that interaction with DC-SIGN could influence the pro-inflammatory response. Lewis negative strains escape binding to DC and induce a strong Th1-cell response. In contrast, *H. pylori* strains that express Le^x^ and/or Le^y^ can bind to DC-SIGN and enhance the production of IL-10 which promotes a Th2-cell response and blocking of Th1-cell activation.

In the context of gastric MALT lymphoma, the chronicity of *H. pylori* infection is believed to be crucial [6]. According to Suarez *et al*., the chronic microbial antigenic stimulation observed during persisting *H. pylori* infection constitutes an antigenic source in autoimmunity which leads to sustained B-cell stimulation, thus favoring lymphoid transformation and lymphoma development [13]. Therefore, in this chronic disease a molecular mimicry, which implies the expression of microbial pathogen motifs shared with the host, could be mandatory to favor *H. pylori* persistence. Lewis determinants resemble autoantigens because of the molecular mimicry with the fucosylated Lewis antigens [14] expressed by the mucosal chief and parietal cells of the gastric glands as well as on the surface and foveolar epithelia [15].

Le^x^ and Le^y^ (type 2 carbohydrates) are the dominant Lewis antigens in *H. pylori* LPS expressed by 80–90% of clinical isolates whereas Le^a^, Le^b^, H-1 (type 1 carbohydrates), Lewis c, and sialyl Le^x^ are rarely expressed (less than 5%) [16], [17]. Three fucosyltransferases FutA, FutB and FutC are involved in Lewis antigen synthesis. FutA and FutB which have an α1,3 and/or α1,4-fucosyltransferase activity are required for Le^x^ or Le^a^ antigen synthesis, respectively. FutC which has an α1,2 fucosyltransferase activity transfers an additional fucose to produce Le^y^ or Le^b^ antigens. The three corresponding genes, ie, *fut*A (HP0379), *fut*B (HP0651) and *fut*C (HP0093-94), contain poly-C tracts at the 5′ end that regulates their expression by a slipped strand mispairing mechanism [18].

The Le^x^ and Le^y^ antigens expressed on the LPS of *H. pylori* gastric MALT lymphoma strains has not yet been investigated. Therefore, the aim of this project was to study these LPS structures of *H. pylori* gastric MALT lymphoma strains in comparison to strains solely associated with chronic gastritis.

## Results

### Description of the *cag* pathogenicity island (*cag*PAI) status and vacuolating cytotoxin gene A (*vac*A) genotypes of the strains included in the present study

As indicated in material and methods, 40 MALT strains (19 *cag*PAI (−) and 21 *cag*PAI (+)) and 39 gastritis strains (17 *cag*PAI (−) and 22 *cag*PAI (+)) were included in this study.

The 39 gastritis strains were classified according to the signal region as either s1 or s2 or according to the middle region as m1 or m2 of the *vac*A gene. The distribution of the different alleles, s1m1, s1m2 and s2m2 of the gastritis strains were 16 (41%), 15 (38.5%) and 8 (20.5%), respectively.

As previously determined [4], the different *vac*A alleles for MALT strains were 11 s1m1 (27.5%), 12 s1m2 (30%) and 17 s2m2 (42.5%).

### Distribution of the Le^x^ and Le^y^ antigens among the strains according to the disease status and virulence factors

Le^x^ was identified in 21 MALT strains (52.5%) and Le^y^ in 30 strains (75%). Le^x^ was identified in 29 gastritis strains (74.3%) and Le^y^ in 31 strains (79.5%). Depending on the Lewis antigens expressed, the strains were divided into four groups. Five gastritis strains (12.8%) and 5 MALT strains (12.5%) were Le^x/y^ negative. Three gastritis strains (7.7%) and 5 MALT strains (12.5%) only expressed Le^x^. Five gastritis strains (12.8%) and 14 MALT strains (35%) solely expressed Le^y^. Finally, 26 gastritis strains (66.7%) and 16 MALT strains (40%) were both Le^x/y^ positive.

There was a significant association between *cag*PAI status and Le^x^ expression among MALT strains (*p*<0.0001), but not in gastritis strains (*p* = 0.64, NS).

There was a significant interaction between Lewis antigens and *cag*PAI status in relation to the disease status (*P* value for interaction = 0.028), therefore we stratified the analysis.

Among *cag*PAI negative strains, Le^x^ were expressed in 12 gastritis strains (70.6%) and only in 3 MALT strains (15.8%); and Le^y^ in 13 gastritis strains (76.5%) and in 13 MALT strains (68.4%). In a regression model, *cag*PAI negative strains expressing solely Le^y^ were associated with MALT with an odds ratio (OR) of 64.2 with 95% confidence intervals (95% CIs) 4.9–841.0 when compared to Le^x/y^ positive strains (Table 1).

**Table 1:** Distribution of the Lewis phenotypes among gastritis and MALT lymphoma strains of *Helicobacter pylori* according to the *cag*PAI status.

Lewis antigens	Gastritis n (%)	MALT n (%)	Odds ratio * (95% confidence interval)
Among *cag*PAI (−)			
Le^x+/y+^	11 (64.7)	1 (5.3)	1.0 (reference)
Le^x+/y−^	1 (5.9)	2 (10.5)	19.9 (0.8–485.0)
Le^x−/y+^	2 (11.8)	12 (63.2)	**64.2** (4.9–841.0)
Le^x−/y−^	3 (17.6)	4 (21.0)	12.9 (0.9–178.2)
Among *cag*PAI (+)			
Le^x+/y+^	15 (68.2)	15 (71.4)	1.0 (reference)
Le^x+/y−^	2 (9.1)	3 (14.3)	2.7 (0.20–37.7)
Le^x−/y+^	3 (13.6)	2 (9.5)	0.69 (0.08–5.6)
Le^x−/y−^	2 (9.1)	1 (4.8)	0.39 (0.03–5.8)

* Odds of being MALT/odds of being gastritis, with adjustment for gender and age.

Among *cag*PAI positive strains, Le^x^ were expressed in 17 gastritis strains (77.3%) and 18 MALT strains (85.7%); and Le^y^ in 18 gastritis strains (81.8%) and 17 MALT strains (81.0%). *cag*PAI positive strains expressing solely Le^y^ were not associated with MALT when compared to Le^x/y^ positive strains (OR 0.4; 95% CI 0.03–5.8) (Table 1).

*vac*A genotypes did not modify the association between Lewis antigens and disease status.

### Sequencing of *fut*A and *fut*B for Le^x/y^ negative strains

The LPS extraction was verified on SDS/PAGE gels after silver staining (data not shown) for the Le^x/y^ negative strains: 5 gastritis strains (3 *cag*PAI (−) and 2 *cag*PAI (+)) (G152, G171, G541, G32 andG447) and 5 MALT lymphoma strains (3 *cag*PAI (−) and 1 *cag*PAI (+)) (M30, M33, M40 and M48). The *fut*A and *fut*B genes were then sequenced. According to the number of C repeats that regulates the on/off status of the genes, 9 strains were *fut*A “off” and one strain *fut*A “on” (M54), 8 strains were *fut*B “off” and 2 strains *fut*B “on” (G447, G541). To summarize, a total of 7 strains were “off” for both the *fut*A and *fut*B genes (ie, M30, M33, M40, M48, G152; G171 and G32), one strain was *fut*A “on” only (M54) and in two strains only *fut*B was “on” (ie, G541 and G447) (Table 2). The number of C repeats varied between 8 to 13 for *fut*A and from 8 to 11 for *fut*B. The first 91 bp of the 5′ part of the genes which contains the CC repeats, as well as the deduced amino acid sequenced, are shown in Table 2.

**Table 2:** Signal-sequence coding region of the *fut*A and *fut*B genes and deduced amino acid sequences of 10 Le^x/y^ negative *Helicobacter pylori* strains.

Loci	Strains	Sequence of the signal-peptide coding region	C repeat	Status	Deduced amino acid sequence
***fut*** **A**	M48	ATGTTCCAGCCCTTACTAGACGCCTTTATAGAAAGCGCTTCCATTGAAAAAATGGCCTCTAAATCTCCCCCCCC-----TAACAATCGCTG	8	Off	MFQPLLDAFIESASIEKMASKSPPPNNRCGELVGR*
	M30	ATGTTTCAGCCCTTACTAGACGCCTTTATAGAAAGCGCTTCCATTGAAAAAATGGCCTCTAAATCTCCCCCCCCC----**TAA**AAATCGCTG	9	Off	MFQPLLDAFIESASIEKMASKSPPP*
	M33	ATGTTCCAGCCCCTATTAGACGCCTTTATAGAAAGCGCTTCAATTAAAAAAATGGCCTCTAAGTTACCCCCCCCC----**TAA**AAATCGCTG	9	Off	MFQPLLDAFIESASIKKMASKLPPP*
	M40	ATGTTCCAGCCCCTATTAGACGCTTTTATAAAAGGCGCTTCCTTTGAAAAAATGGCCTCTAAGTTACCCCCCCCC----**TAA**AAATCGCTG	9	Off	MFQPLLDAFIKGASFEKMASKLPPP*
	G152	ATGTTCCGCCCCTTACAAAACCCTTTTATAAAAGGCGCTTCCTTTGAAAAAAGGGTTTCTAAATCTCCCCCCCCC----**TAA**AAATCGCTG	9	Off	MFRPLQNPFIKGASFEKRVSKSPPP*
	G171	ATGTTCCAGCCCCTATTAGACGCCTTTATAGAAAGCGCTTCCATTGAAAAAATGGTCTCTAAATCTCCCCCCCCC----**TAA**AAATCGCTG	9	Off	MFQPLLDAFIESASIEKMVSKSPPP*
	G541	ATGTTTCAGCCCTTACTAGACGCCTTTATAGAAAGCGCTTCCATTGAAAAAATGGCCTCTAAATCTCCCCCCCCC----**TAA**AAATCGCTG	9	Off	MFQPLLDAFIESASIEKMASKSPPP*
	G32	ATGTTTCAGCCCTTACTAGACGCCTTTATAGAAAGCGCTTCCATTGAAAAAATGGTTTCTAAATCTCCCCCCCCCCC--TAAAAATCGCTG	11	Off	MFQPLLDAFIESASIEKMVSKSPPPPKNRCGELVGR*
	G447	ATGTTCCAACCCTTACTAGACGCCTTTATAGAAAGCGCTTCCATTGAAAAAATGGCCTCTAAATCTCCCCCCCCCCC--TAAAAATCGCTG	11	Off	MFQPLLDAFIESASIEKMASKSPPPPKNRWGELGGR*
	M54	ATGTTCCAACCCTTACTAGACGCCTTTATAGAAAGCGCTTCCATTGAAAAAATGGCCTCTAAATCTCCCCCCCCCCCCCTAAAAATCGCTG	13	On	MFQPLLDAFIESASIEKMASKSPPPPLKIAVANWWG
***fut*** **B**	M30	ATGTTCCAACCCCTATTAGACGCCTTTATAGAAAGCGCTTCCATTGAAAAAATTACCTCTAAATCTCCCCCCCC-----TAAAAATCGCTG	8	Off	MFQPLLDAFIESASIEKITSKSPPPKNRCGELVGR*
	M48	ATGTTCCAGCCCTTACTAGACGCCTTTATAGAAAGCGCTTCCATTGAAAAAATGGCCTCTAAATCTCCCCCCCC-----TAACAATCGCTG	8	Off	MFQPLLDAFIESASIEKMASKSPPPNNRCGELVGR*
	M33	ATGTTCCAGCCCCTATTAGACGCCTTTATAGAAAGCGCTTCAATTAAAAAAATGGCCTCTAAGTTACCCCCCCCC----TAAAAATCGCTG	9	Off	MFQPLLDAFIESASIKKMASKLPPP*
	M40	ATGTTCCAACCCCTATTAGACGCCTTTATAGAAAGCGCTTCCATTGAAAAAATTACCTTTAAATCTCCCCCCCCC----**TAA**AAATCGCTG	9	Off	MFQPLLDAFIESASIEKITFKSPPP*
	M54	ATGTTCCAGCCCCTATTAGACGCCTTTATAGAAAGCGCTTCCATTGAAAAAATGGCCTCTAAATCTCCCCCCCCC----**TAA**AAATCGCTG	9	Off	MFQPLLDAFIESASIEKMASKSPPP*
	G152	ATGTTCCAGCCCCTACTAGACGCCTTTATAGAAAGCGCTTCCATTGAAAAAATGGCCTCTAAATCTCCCCCCCCC----**TAA**AAATCGCTG	9	Off	MFQPLLDAFIESASIEKMASKSPPP*
	G171	ATGTTCCAGCCCCTATTAGACGCCTTTATAGAAAGCGCTTCCATTGAAAAAATGGTCTCTAAATCTCCCCCCCCC----**TAA**AAATCGCTG	9	Off	MFQPLLDAFIESASIEKMVSKSPPP*
	G447	ATGTTCCAACCCCTATTAGACGCCTTTATAGAAAGCGCTTCCATTGAAAAAATGGCCTCTAAATCTCCCCCCCCCC---TAAAAATCGCTG	10	On	MFQPLLDAFIESASIEKMASKSPPPLKIAVANWWGD
	G541	ATGTTCCAGCCCCTATTAGACGCCTTTATAGAAAGCGCTTCCATTGAAAAAATGGCCTCTAAATCTCCCCCCCCCC---TAAAAATCGCTG	10	On	MFQPLLDAFIESASIEKMASKSPPPLKIAVANWWGD
	G32	ATGTTCCAACCCTTACTAGACGCCTTTATAGAAAGCGCTTCCATTGAAAAAATGGCCTTTAAATCTCCCCCCCCCCC--TAAAAATCGCTG	11	Off	MFQPLLDAFIESASIEKMAFKSPPPPKNRCGELVGR*

G: gastritis strain. M: MALT strain. The stop codon is indicated in bold type for strains having the “off” status (except for strains M48, G32 for the *fut*A locus, and M30, M48, M33 and G32 for the *fut*B locus) and the end of the protein is indicated by an asterisk.

## Discussion

The LPS expressed by *H. pylori* strains has been suggested to be important for gastric colonization, adherence and immune evasion through a “camouflage” mechanism in order to escape the host immune response [19]. The influence of Lewis antigens is also believed to participate in the polarization of the Th1/Th2 inflammatory response. Lewis antigens expressed by *H. pylori* MALT strains could play a key role in the physiopathology of the disease. We previously described the main virulence factors expressed by gastric MALT lymphoma strains except the Lewis antigens [4]. Therefore, in the present study, the Lewis antigen expression was compared between *H. pylori* MALT strains and the chronic gastritis strains. The gastritis strains were selected on the presence or absence of the *cag*PAI in order to match the gastric MALT lymphoma strains and to constitute two comparable groups.

Most of the strains (59/69, 85.5%) included in the present study expressed at least one Lewis antigen (Le^x^ and/or Le^y^) which is in line with previous reports [17]. Although it is well known that a combination of all several methods (serodot, Western blotting and enzyme-linked immunosorbent assay) may allow a more accurate assessment of Le expression [20], only 10 double Le^x/y^ negative strains were identified and mainly among *cag*PAI negative strains (60%) as already described. Concerning these double negative strains and according to the number of C repeats, the correlation with the *fut*A and *fut*B sequences was not perfect. Indeed, because 3 of these 10 strains were “on” for *fut*A or *fut*B, we cannot exclude that they do not express some of the other less common Lewis antigens [16].

The Lewis antigen patterns were then analyzed according to the *cag*PAI status and *vac*A genotypes of the strains. This is an issue that has been extensively discussed previously. A first study published by Wirth *et al.* showed that Le^x^ expression in *H. pylori* was correlated with its CagA status [21]. Some studies suggest that such a correlation does not exist [22] or that an inverse correlation exists between CagA positive strains and Le^x^ and Le^y^ expression [23]. Broutet *et al.* studied the LPS of 155 isolates from atrophic gastritis patients, and identified two main clusters of strains, those which were CagA positive and double Le^x/y^ positive, and a second cluster comprised of CagA negative and solely Le^y^ positive or double Le^x/y^ negative [24]. The authors also showed a correlation with *vac*A genotypes. However, more recently, Skoglund *et al.*, considering only Le antigen expression, were not able to confirm such a correlation for atrophic gastritis isolates [25]. Nevertheless, CagA positive isolates are believed to be more aggressive and more exposed to the immune system. Therefore, a camouflage by an efficient mimicry process could help them to persist in the stomach of their host. A similar association was found in the present study for Le^x^ and *cag*PAI positive gastric MALT lymphoma strains. Indeed, the proinflammatory properties of *cag*PAI positive gastric MALT lymphoma strains have been previously proven using the AGS cell model (most *cag*PAI positive MALT lymphoma strains harbored a functional *cag*PAI) but no important proinflammatory factors could be identified in *cag*PAI negative *H. pylori* MALT strains [26]. According to Moran, all of the discrepancies between Lewis antigens expression and *cag*PAI status are believed to be due to the adaptation of *H. pylori* strains to different populations [19], [27]. However, in contrary to the study of Broutet *el al*., the present study did not identify a correlation between Lewis antigens and *vac*A genotypes [24].

Concerning the disease status among *cag*PAI negative MALT strains, a significant association was found with Le^y^. Although we were not able to quantify the amount of Le^y^ expressed by the strains, the Le^y^ bands obtained by Western Blot were very intense (data not shown). It has been suggested that a high expression of Le^y^ could mask the Le^x^ epitopes [18]. The activity of *fut*C in Le^y^ synthesis is very critical. Indeed, strains that have a relatively high level of *fut*C activity could transform nearly all of the Le^x^ to Le^y^ giving rise to a phenotype expressing solely Le^y^, whereas relatively lower activity is believed to be responsible for a simultaneous Le^x^ and Le^y^ phenotype. Like *fut*A and *fut*B, *fut*C is regulated by a slipped-strand mispairing mechanism through a polyC tract as well as an imperfect TAA repeat in the mid-region of *fut*C [28]. The *fut*C expression could also be influenced by translational frameshift, transcriptional regulation of certain genes as well as deletion in the promoter region. Therefore, we believe that the high Le^y^ expression in *cag*PAI negative MALT strains should be studied more extensively on a genetic basis.

What could the role of Le^y^ be in *cag*PAI negative MALT strains? During *H. pylori* chronic infection, Le^y^ expression has been implicated in the pathogenesis of atrophic gastritis through the induction of autoreactive antibodies against the gastric mucosa [19]. For example, such antibodies have been found in patients with atrophic gastritis and gastric cancer. Skoglund *et al.* recently published a paper in which they showed that the HPAG1 strain (which is a Le^y^ positive strain) was able to switch *in vivo* to both Le^x^ and Le^y^ expression, while the same strain in a mouse model of gastric atrophy remained to exclusively express Le^y^. The authors concluded that the switch in Lewis antigen expression was linked to different gastric environments, and possibly gastric pH. However, the authors were not able to reproduce this phenomenon *in vitro* by using culture broth at different pH [25]. Indeed, it has been shown that *fut*C transcription can be regulated by environmental factors such as gastric acid and host immunity [29]. The study of Skoglund *et al.* indicates that Lewis expression at the time of isolation can be considered as a “snap-shot” of a strain at a particular time of the disease status/development reflecting the bacterial adaptation to *in vivo* conditions. Therefore, the higher expression of Le^y^ in *cag*PAI negative MALT strains identified in the present study raises the question of the presence of a component of atrophy in patients infected by these types of strains. This particular point has been suggested in long-term infected BALB/c mice [30], [31]. The presence of auto anti Le^y^ antibodies in the physiopathology of this disease should also be evaluated.

In the context of gastric MALT lymphoma, intra Th lymphocytes are essentially Th0, and Greiner *et al.* showed the important role of cytokines produced by Th2 lymphocytes (IL-4, IL-10) [32]. Similarly, animal models for MALT lymphoma (BALB/c mouse) showed a Th2 type response [31], [33]. Because most of the MALT strains expressed at least one Lewis antigen, they should be able to polarize a Th2 inflammatory response and to address this major point an *in vitro* study is currently being performed using DCs.

Our study shows for the first time that gastric MALT lymphoma strains, especially *cag*PAI negative, can be distinguished from gastritis strains. This particular Lewis profile is probably representative of the adaptation of *H. pylori* during the micro-environmental changes that the bacterium encounters in the course of infection. Moreover, it could represent an adaptive mechanism to the host response that should be explored *in vivo* in a murine model of gastric MALT lymphoma, and especially on a long-term survival in the host and modulation of host immune responses (autoimmune antibody production, release of cytokines).

## Materials and Methods

### Bacterial strains

A subset of the French gastric MALT lymphoma strain collection provided by the French National Reference Center for Campylobacters and Helicobacters was included in this study: 40 strains from gastric MALT lymphoma patients (24 men and 16 women), mean age 58.4 (13.8)). For all these strains the *cag*PAI status and *vac*A genotypes have been previously published: 19 *cag*PAI (−) and 21 *cag*PAI (+) [4].

A selection of 39 strains isolated from chronic gastritis only patients (16 men and 23 women, mean age 48.3 (13.2)) were included as a population control. All of these strains were isolated from patients included in the Swedish Kalixanda study which was performed on a general adult population from northern Sweden. The strains included in this study were selected according to their *cag*PAI status (17 *cag*PAI (−) and 22 *cag*PAI (+)) in order to be comparable with the MALT strain collection [34].

### LPS purification

All of the strains (previously obtained from single colonies) were defrosted on G plates before LPS extraction. Then *H. pylori* was grown in brucella broth supplemented with 5% fetal bovine serum and 1% IsoVitaleX Enrichment to exponential phase (OD_600nm_ between 0.3 and 0.6). An equal amount of bacteria from each strain was harvested by centrifugation and washed once in PBS. The LPS from each bacterial pellet were extracted twice using a hot phenol protocol as already described [18]. After an overnight precipitation in ethanol and sodium acetate, the LPS were dried in air and resuspended in water.

### Polyacrylamide gel electrophoresis and immunobloting

LPS extracts were analyzed by SDS/PAGE by using a 4–15% separating polyacrylamide gel and blotted onto a polyvinylidene difluoride membrane (Immun-Blot PVDF Membrane, Biorad, Hercules, CA). Mouse anti-Le^x^ (MCA 1313, Serotec, dilution 1/500) and anti-Le^y^ (BG-8, Signet Laboratories Inc., Dedham, MA, dilution 1/2000) were used as primary antibodies and horseradish peroxidase-conjugated goat anti-mouse IgM (Start 86P, Serotec, dilution 1/1000) as a secondary antibody. Membranes were incubated with antibodies and developed with enhanced chemiluminescence (ECL) (Amersham Pharmacia Biosciences, Buckinghamshire, UK.) as already described [18], [25], [35]. The LPS extraction for Le^x^ and Le^y^ negative strains was also verified on SDS/PAGE gels stained with silver.

### PCR and sequencing

*H. pylori* DNAs were purified with DNeasy Tissue Kit (Qiagen, Hilden, Germany) according to the manufacturer's recommendations from bacterial pellets obtained from the same brucella broth as those used for LPS extraction. For gastritis strains, the *vac*A alleles (s and m regions) were detected by PCR as previously described [4], [36]. Finally, the 5′end parts of genes encoding FutA and FutB were amplified and sequenced for Le^x^ and Le^y^ negative gastritis and MALT strains identified in this study. The sequences of the primers used for *fut*A and *fut*B amplification and sequencing are indicated in Table 3.

**Table 3:** Sequences of the primers used to amplify and/or sequence *fut*A and *fut*B genes isolated from *Helicobacter pylori* gastric MALT lymphoma and chronic gastritis strains.

Primer	Sequence 5′-3′	Target	use
***fut*** **A-F**	CTCTCGTGATCTTGGCTTATT	*fut*A^1^	PCR and sequencing
***fut*** **A-R**	AAGTAGCGTCTGCGATGA	*fut*A^1^	PCR and sequencing
***fut*** **B-F**	GCCCTAATCAAGCCTTTG	*fut*B^1^	PCR and sequencing
***fut*** **B-R**	AAAACCCCACGCTCAAAAA	*fut*B^1^	PCR and sequencing
***fut*** **A-** ***fut*** **B-F**	TTCCAACCCCTATTAGACG	*fut*A and *fut*B^2^	Sequencing
***fut*** **A-** ***fut*** **B-R**	TTCTCACACTTCCTCCCCC	*fut*A and *fut*B^2^	Sequencing

1 indicates that the primer binding sequence is located outside of the coding region of the genes.

2 indicates that the primer binding sequence is located inside of the coding region of the genes.

F for a forward primer; R for a reverse primer.

### Statistical methods

All analyses were conducted using the SAS 9.2 package (SAS Institute, Cary, NC). Correlations between genes were tested by the Spearman method. ORs with 95% CIs derived from unconditional logistic models were used to assess associations between Lewis antigens and disease status, and *vac*A genotypes and disease status, with adjustments for gender and age. Interaction effects between and among genotypes and Lewis antigens were tested by inclusion of product terms in regression models.
